# Supplementary material for: A dendritic disinhibitory circuit mechanism for pathway-specific gating
Source: Nat Commun. 2016 Sep 20;7:12815. doi: 10.1038/ncomms12815 (PMC5034308; doi:10.1038/ncomms12815)
Supplement: Supplementary Information — Supplementary Figures 1-8, Supplementary Tables 1-2, Supplementary Notes 1-2 and Supplementary References. [file ncomms12815-s1.pdf]

Supplementary Figures

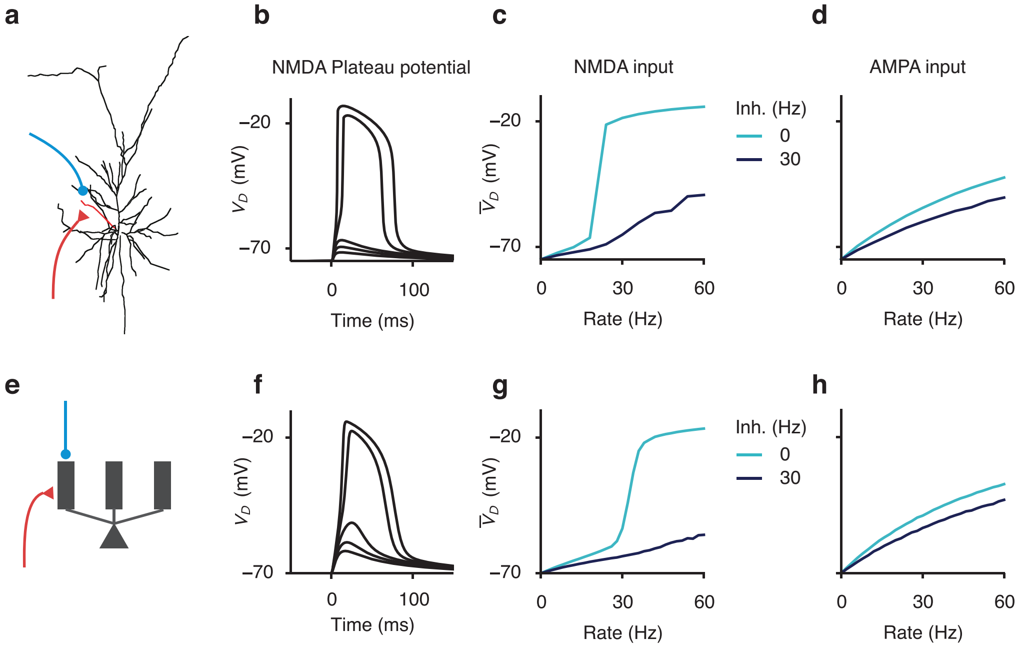

Supplementary Figure 1. Dendritic disinhibition powerfully gates dendritic nonlinearity.

(**a-d**) Dendritic disinhibition controls NMDAR-dependent nonlinearity in a reconstructed compartmental neuron model. (**a**) A morphologically reconstructed compartmental model of a layer 2/3 pyramidal neuron (1) receives excitatory and inhibitory inputs uniformly distributed onto one basal dendrite. (**b**) Excitatory inputs can generate a local, regenerative NMDA plateau potential in the dendrite. As the number of activated synapses is increased, there is a sharp nonlinear increase in the evoked dendritic membrane depolarization (V_D). (**c-d**) Presynaptic spike times are modeled as Poisson-distributed events. (**c**) In response to synaptic input mediated by NMDAR channels, the mean dendritic voltage across time ($\overline{V}_D$) increases nonlinearly as a function of excitatory rate (light blue). Moderate inhibition largely suppresses NMDA plateau potentials even for high excitatory input rate (dark blue). (**d**) The effect of inhibition is much weaker when excitatory input is mediated by AMPARs. 20 excitatory synapses are used as input in (**c,d**). (**e-h**) A reduced compartmental neuron model captures the nonlinearity of the morphologically reconstructed model. (**e**) A somatic compartment is connected to multiple, otherwise independent, dendritic compartments (only three shown). (**f-g**) Modeling results in the reconstructed neuron model (**b-d**) are reproduced by the simplified model. 15 excitatory synapses are used as input in (**g,h**).

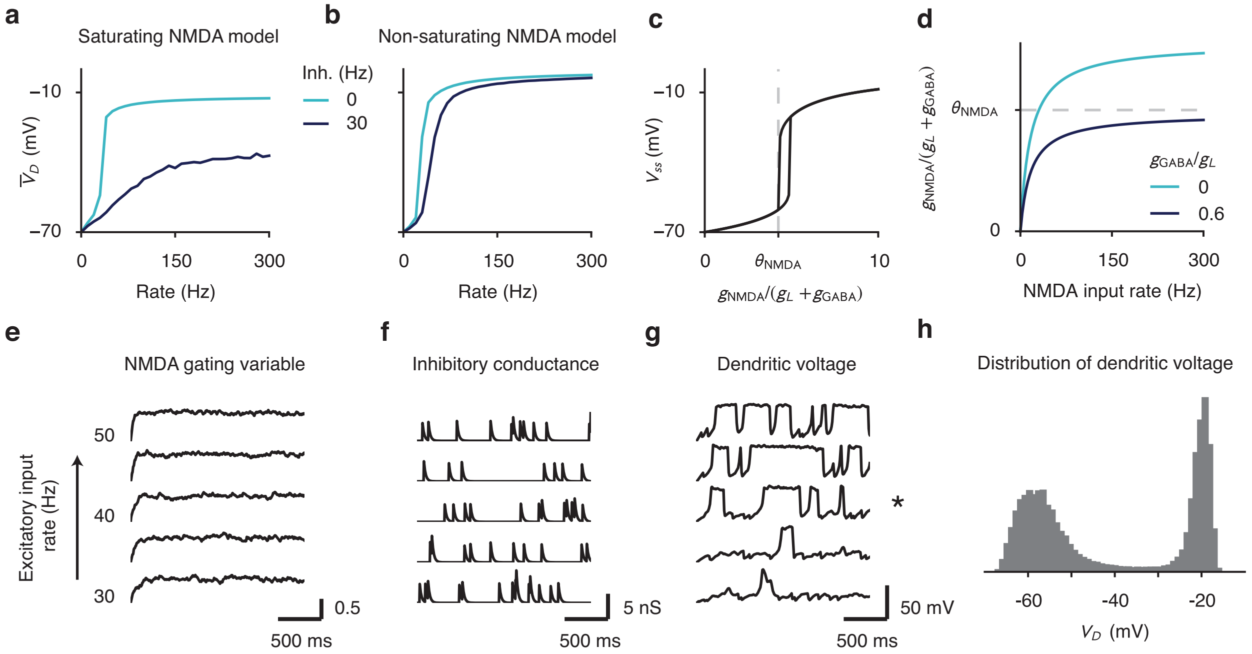

Supplementary Figure 2. Effects of NMDA receptor saturation and low-rate inhibition. (**a-d**) NMDAR saturation allows for much stronger inhibitory control. (**a**) Using an NMDAR model with saturation allows mild dendritic inhibition to powerfully control dendritic voltage. Note that voltage of the inhibited dendrite (dark blue) never reaches the same level as the disinhibited dendrite (light blue). (**b**) The same level of inhibition has a much smaller effect when we used a non-saturating NMDAR model. (**c**) For constant synaptic conductance, the steady-state voltage of one dendritic branch (V_{ss}) increases sharply with the effective input $g_{NMDA}/(g_L + g_{GABA})$, where g_{NMDA}, g_L, and g_{GABA} are the NMDAR, leak, and GABAR conductances, respectively. The dashed line indicates the threshold θ_{NMDA} below which V_{ss} is stably in the low state. (**d**) The NMDR conductance, and therefore $g_{NMDA}/(g_L + g_{GABA})$, saturates at high input rates to NMDAR synapses. With moderate inhibition, the saturated value of the effective input can be lower than the threshold θ_{NMDA} for an NMDA plateau potential. (**e-h**) Low-rate (temporally sparse) Poisson inhibition generates irregular NMDA plateau potentials and graded encoding of input rate. Inhibition is said to be temporally sparse when the product of the inhibition rate r_I and the time constant τ_{GABA} of GABAR is much smaller than 1, i.e. $r_I \cdot \tau_{GABA} \ll 1$ (**e**) Due to relatively high input rate and long time constant, the NMDAR gating variable averaged across synapses is nearly constant in time. Each trace corresponds to a different excitatory input rate, ranging from 30Hz (bottom) to 50Hz (top); the same applies to (**f,g**). (**f**) Inhibitory conductance is temporally sparse due to a low background inhibition rate of 5 Hz. (**g**) The dendritic voltage switches stochastically in time, into and out of the NMDA plateau potential. (**h**) The dendritic voltage across time exhibits a bimodal distribution, due to stochastic switching. The excitatory rate is set to 40 Hz (asterisk in (**g**)).

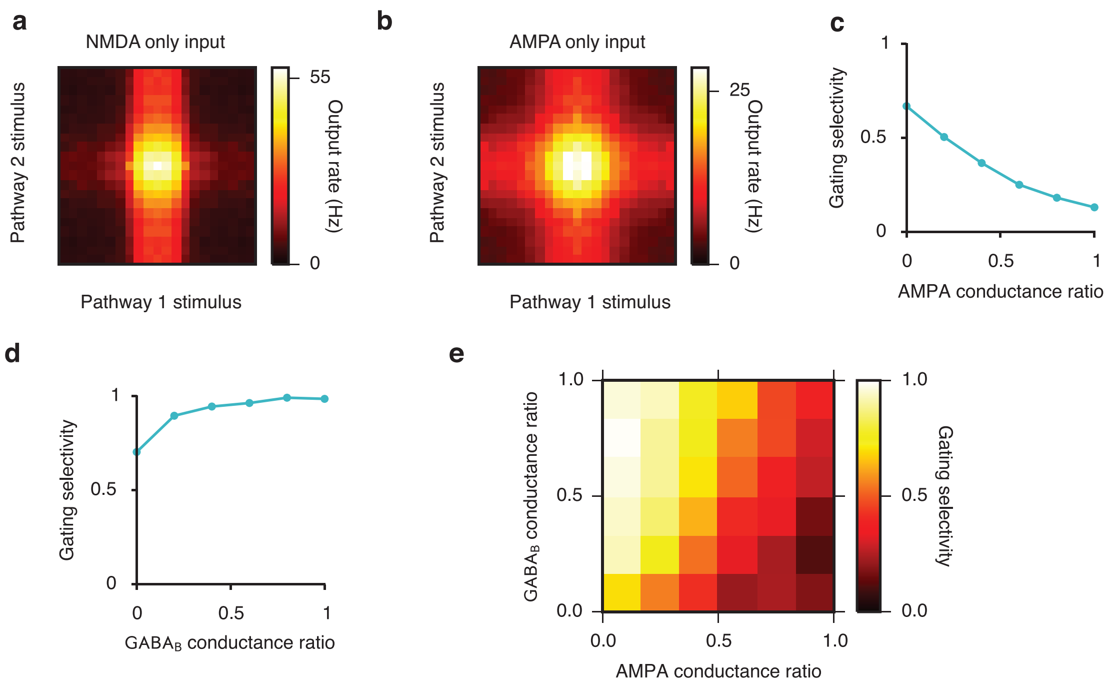

Supplementary Figure 3. Pathway-specific gating with varying levels of AMPAR and GABA$_B$ conductance. In the majority of our work, dendritic excitation is mediated only by NMDARs and dendritic inhibition only by GABA$_B$Rs. Here we show how pathway-specific gating varies with the inclusion of AMPAR and GABA$_B$ inputs. (**a**) Pathway-specific gating when excitatory input is mediated solely by NMDARs, adapted from **Fig. 2g** for comparison. (**b**) When the excitatory input is conducted solely by AMPARs (maximum conductance $\tilde{g}_{\mathrm{AMPA}}$ =2.5 nS for each synapse), the gating performance is strongly degraded. All other conditions are kept the same in (**a**) and (**b**). Disinhibited dendrites receive 30-Hz disinhibition. (**c**) Gating selectivity (which ranges from 0 for no gating to 1 for perfect gating, see Experimental Procedures for the definition) decreases as a function of the AMPA conductance ratio. Here AMPA conductance ratio is defined as $\tilde{g}_{\mathrm{AMPA}}/(\tilde{g}_{\mathrm{AMPA}}+\tilde{g}_{\mathrm{NMDA}})$, which is 0 in the NMDAR-only case and 1 in the AMPAR-only case. $\tilde{g}_{\mathrm{AMPA}}+\tilde{g}_{\mathrm{NMDA}}$ is held constant at 2.5 nS. (**d**) Gating selectivity increases as a function of the GABA$_B$ conductance ratio. This is due to both the slower dynamics of GABA$_B$ receptors and the inward-rectifying potassium (KIR) conductance activated by GABA$_B$ receptors (2; 3). Here excitatory inputs are mediated by NMDARs only. (**e**) Gating selectivity remains high for a wide range of combinations of AMPA and GABA$_B$ conductance ratio.

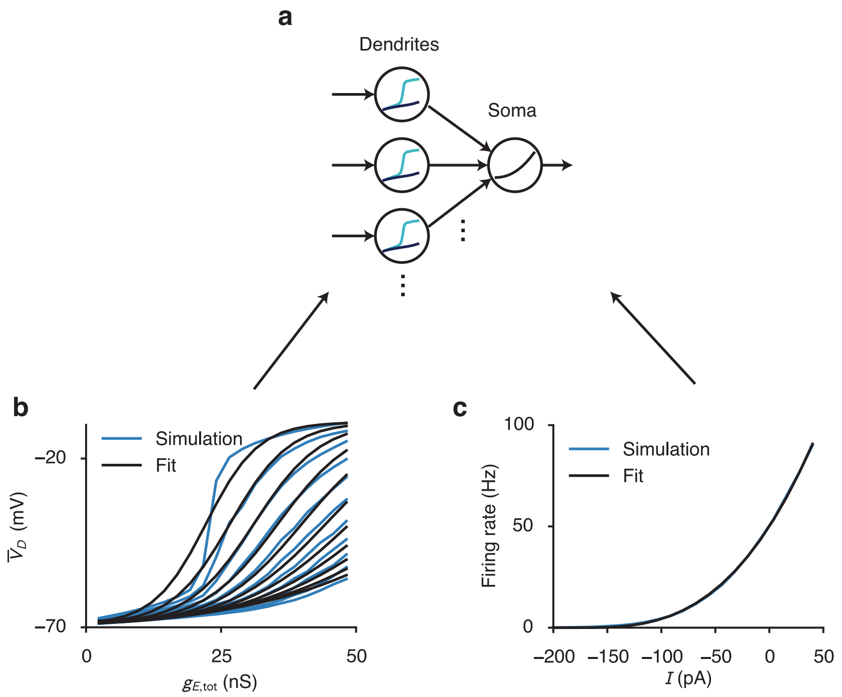

Supplementary Figure 4. Multi-compartment rate model for pyramidal neurons based on the reduced spiking neuron model. (**a**) The neuron model is comprised of multiple dendrite compartments, whose mean voltages are modeled with a family of sigmoidal functions. These dendritic voltages are converted into currents and fed into a somatic compartment, whose firing rate output is modeled with a power-law function. (**b**) The mean dendritic voltage ($\overline{V}_D$) as a function of excitatory and inhibitory inputs. (Blue) Simulation of the reduced-compartmental spiking neuron model. 15 NMDAR inputs fire at a Poisson rate of 30 Hz with conductance ranging from 0.25 to 5.0 nS, resulting in total conductance ($\overline{g}_E$) approximately between 0 and 50 nS. Each curve corresponds to a different inhibitory input rate, ranging uniformly from 0 Hz (top curve) to 100 Hz (bottom curve), in increment of 10 Hz. (Black) Fit of the simulation results. All curves are simultaneously fit with a family of sigmoidal functions, where parameters of the sigmoid, i.e. mid-point and width, are controlled by inhibition. The back-propagating action potential is fixed at a rate of 10 Hz. (**c**) Somatic firing rate as a function of input current from dendrites (and potentially PV neurons). In our model, since at resting state the mean dendritic voltage is lower than the somatic voltage, the input current is negative. The simulation result of the spiking model (Blue) is fit with a power-law function (Black).

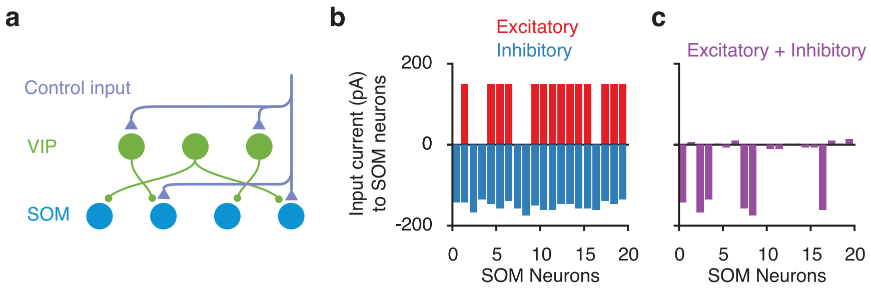

Supplementary Figure 5. Mechanism of control. (**a**) In this scenario, we assume that for each pathway control inputs target a random subset of VIP and SOM neurons. (**b-c**) Input currents onto SOM neurons (only 20 shown). (**b**) 50% of the SOM neurons receive excitatory currents from control (red). 50% of VIP neurons receive excitatory control, but due to the high random connectivity from VIP to SOM neurons, inhibitory currents onto SOM cells are nearly uniform (blue). (**c**) The sum of the excitatory and inhibitory currents onto SOM neurons, i.e. the total currents, are primarily inhibitory and vary strongly across SOM neurons. The overall inhibitory currents are results of overall stronger inhibition. The variability across SOM neurons are mainly inherited from the selective excitatory control input.

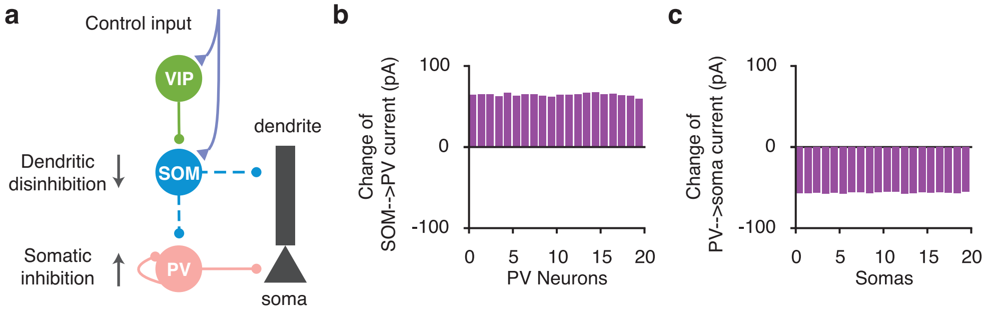

Supplementary Figure 6. Inclusion of PV neurons results in a uniform somatic inhibition across pyramidal neurons. (**a**) SOM suppression lead to PV disinhibition and somatic inhibition. Same as **Fig. 6a**. (**b**) The change in the SOM-to-PV input currents after the control input. The change in currents is disinhibitory (net excitatory) with a small standard deviation compared to the mean across PV neurons. Notice that although the control input results in a selective suppression of SOM neurons (**Supplementary Figure 5c**), the change in the SOM-to-PV currents is almost uniform due to the high SOM-to-PV connection probability. (**c**) The change in the PV-to-soma input currents after the control input is net inhibitory and again uniform across somas. Therefore a selective suppression of SOM neurons results in a non-selective inhibition across somas through PV neurons.

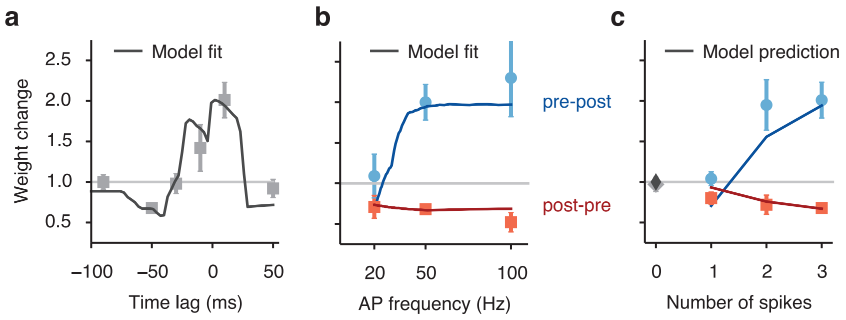

Supplementary Figure 7. Fit and prediction of the plasticity model compared to experimental data. (**a**-**c**) A pre-synaptic spike is paired with multiple post-synaptic action potentials (AP). Light symbols mark data showing synaptic weight change (weight after learning/weight before learning) when varying the pre-post time lag (**a**), post-synaptic AP frequency (**b**), and number of post-synaptic spikes (**c**). In (**b**,**c**), the presynaptic spike either precedes (blue) or follows (red) the postsynaptic spikes. Curves in (**a**-**b**) show the model fit, with the same set of parameters. (**c**) The model generalizes to predict data not used to fit the model. Experimental data are extracted from (4).

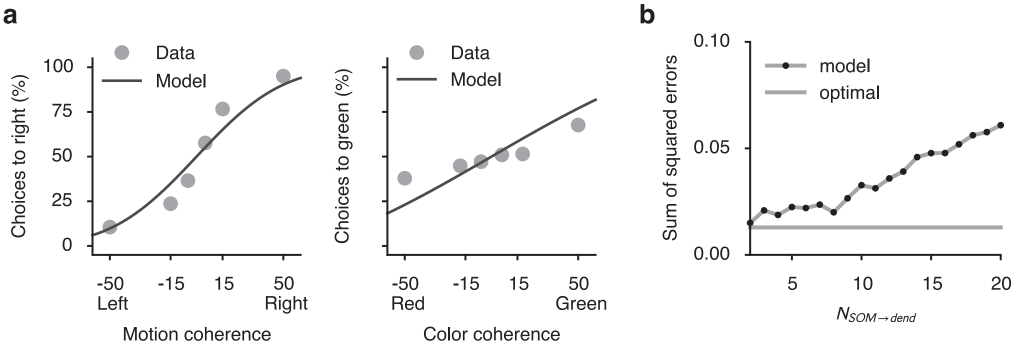

Supplementary Figure 8. Fits of behavioral data as we vary parameters of the interneuronal circuit. (**a**) The model fit to behavioral data in motion context when we set $N_{\text{SOM}\to\text{dend}} = 20$. The fit is much degraded compared to **Fig. 8**. From **Fig. 4** we know that $N_{\text{SOM}\to\text{dend}}$ is the critical parameter for gating selectivity measured on the neural level. (**b**) The sum of squared errors of the model fit as a function of $N_{\text{SOM}\to\text{dend}}$. For a large range of $N_{\text{SOM}\to\text{dend}}$, the model can nearly fit the data optimally. The fit starts to degrade when $N_{\text{SOM}\to\text{dend}} > 10$. Dashed line indicates the error level of the optimal sigmoidal fit, where data are directly fitted to logistic functions. The sum of squared errors shown here is the median error of 50 different model realizations and fits.

Supplementary Tables

Parameter	Value	References	Layer	Area	Animal
Proportion of SOM neurons among inhibitory neurons	0.208	(5)	L2/3	S1	mouse
Proportion of VIP neurons among inhibitory neurons	0.2	(5)	L2/3	S1	mouse
Total number of inhibitory neurons in a column	676 ± 116	(6)	L2/3	S1	rat
Baseline activity of SOM neurons	6.2 ± 0.7 Hz	(7)	L2/3	S1	mouse
Unitary IPSQ from SOM to pyramidal neurons	1.5 ± 0.3 pC	(8)	L2/3	V1	mouse
Unitary IPSQ from VIP to SOM neurons	0.69 ± 0.33 pC	(8)	L2/3	V1	mouse
Connection probability from SOM to pyramidal neurons (within 200μm)	0.71 ± 0.03	(9)	L2/3	frontal cortex	mouse
Connection probability from VIP to SOM neurons (within $25 - 100\mu$m)	0.625 ± 0.12	(8)	L2/3	V1	mouse
Number of basal dendrites on each pyramidal cell (number of total tips)	28.8 ± 2.4	(10)	L2/3	V1	rat
Number of basal dendrites on each pyramidal cell (maximum branches at fixed radius)	20 ± 2.6	(11)	L3	V1	monkey
Number of basal dendrites on each pyramidal cell (maximum branches at fixed radius)	34.2 ± 4.9	(11)	L3	anterior cingulate cortex	monkey

Supplementary Table 1. Raw experimental data used to constrain the VIP-SOM-pyramidal disinhibitory circuit. The error estimates are also taken from the references when available. Some of the data are extracted from their figures since the value is not reported in texts. Specifically, the proportion of VIP neurons is inferred from the proportion of 5HT3a neurons among interneurons and proportion of VIP neurons among 5HT3a neurons.

Model	Figure
Fully-reconstructed spiking pyramidal neuron model	**Supplementary Figure 1a-d**
Reduced multi-compartmental spiking pyramidal neuron model	**Fig. 2,7**, **Supplementary Figure 1e-h,2,3**
Multi-compartmental rate pyramidal neuron model	**Fig. 3-6,8**, **Supplementary Figure 4-6,8**
Rate SOM neurons	**Fig. 4-6,8**, **Supplementary Figure 5,6,8**
Rate VIP neurons	**Fig. 5,6,8**, **Supplementary Figure 5,6,8**
Rate PV neurons	**Fig. 6**, **Supplementary Figure 6**
Calcium-based synaptic plasticity	**Fig. 7**, **Supplementary Figure 7**

Supplementary Table 2. All types of models used, and their corresponding result figures.

Supplementary Notes

1. Gating selectivity critically depends on $N_{\mathrm{SOM}\to\mathrm{dend}}$

The gating selectivity is defined as the mean gating selectivity across neurons,

$$\text{Gating selectivity} = \mathrm{E}_{\text{neuron}}\left[\frac{r_{\text{on}} - r_{\text{off}}}{r_{\text{on}} + r_{\text{off}}}\right] \tag{1}$$

For each neuron, the neural activity given the gated-on pathway is

$$\tilde{r}_{\text{on}} = f_r(\langle \overline{V}_{D,\text{on}} \rangle) \tag{2}$$

, where $\langle \overline{V}_{D,\text{on}} \rangle$ is the mean dendritic voltage across all the dendrites on that neuron for the gated-on pathway. Notice here for simplicity we used an input-output formulation for the somatic compartment that is slightly different from the one used in the main text (the results are the same)

$$r = f_r(\langle \overline{V}_D \rangle) = r_0 + \left(\frac{\langle \overline{V}_D \rangle - E_L}{V_r}\right)^{n_r} \tag{3}$$

After correcting for the baseline, we have

$$r_{\text{on}} = f_r(\langle \overline{V}_{D,\text{on}} \rangle) - f_r(E_L) \tag{4}$$

$$= \left(\frac{\langle \overline{V}_{D,\text{on}} \rangle - E_L}{V_r}\right)^{n_r} \tag{5}$$

Similarly

$$r_{\text{off}} = \left(\frac{\langle \overline{V}_{D,\text{off}} \rangle - E_L}{V_r}\right)^{n_r} \tag{6}$$

So

$$r_{\text{off}}/r_{\text{on}} = \left[(\langle \overline{V}_{D,\text{off}} \rangle - E_L)/(\langle \overline{V}_{D,\text{off}} \rangle - E_L)\right]^{n_r} \tag{7}$$

In the limit of large number of dendrites on each pyramidal neuron, we can replace the averaged dendritic voltage with its expectation over dendrites $\mathrm{E}_{\mathrm{D}}[\cdot]$.

$$\langle \overline{V}_{D,\text{on}} \rangle \approx \mathrm{E}_{\mathrm{D}}\left[\overline{V}_{D,\text{on}}\right] \tag{8}$$

Under this approximation, r_{on} and r_{off} would be the same for every neuron, therefore we have

$$
\begin{aligned}
\text{Gating selectivity} &= \frac{r_{\text{on}} - r_{\text{off}}}{r_{\text{on}} + r_{\text{off}}} && (9)\\
&= -1 + 2/\left(1 + \left[(\mathrm{E_D}[\overline{V}_{D,\text{off}}] - E_L)/(\mathrm{E_D}[\overline{V}_{D,\text{on}}] - E_L)\right]^{n_r}\right) && (10)\\
& && (11)
\end{aligned}
$$

Since dendritic voltage is determined by the total excitatory and inhibitory conductance received,

$$
\begin{aligned}
\overline{V}_D &= f_V(\overline{g}_E, \overline{g}_I) && (12)\\
&= 30 \cdot \left[1 + \tanh\left(\frac{\overline{g}_E - g_{1/2}}{\beta}\right)\right] + V_0 + E_L && (13)\\
&= 30 \cdot \left[1 + \tanh\left(\frac{\overline{g}_E - b_g \cdot (g_{L,D} + \overline{g}_I)}{k \cdot \exp(\overline{g}_I/\gamma)}\right)\right] + V_0 + E_L && (14)\\
& && (15)
\end{aligned}
$$

Remember that for each pathway, we assume that the excitatory input conductance is a deterministic function of the inhibitory conductance received when the corresponding gate is open.

$$
\overline{g}_E = \begin{cases} (1 - \overline{g}_I/g_{I,\text{th}}) \cdot g_{E,\max} & , \overline{g}_I < g_{I,\text{th}} \\ 0 & , \overline{g}_I \geq g_{I,\text{th}} \end{cases} \quad (16)
$$

Denote this rectified linear function as $\overline{g}_E = f_E(\overline{g}_I)$. For convenience consider two pathways, the inhibitory conductance for gate 1 is $\overline{g}_{I,1}$ and for gate 2 is $\overline{g}_{I,2}$. And excitatory conductance for pathway 1 and 2 are $\overline{g}_{E,1} = f_E(\overline{g}_{I,1})$ and $\overline{g}_{E,2} = f_E(\overline{g}_{I,2})$ respectively. Then

$$
\begin{aligned}
\mathrm{E_D}\left[\overline{V}_{D,\text{on}}\right] &= 30 \cdot \left[1 + \mathrm{E_D}\left[\tanh\left(\frac{f_E(\overline{g}_{I,1}) - b_g \cdot (g_{L,D} + \overline{g}_{I,1})}{k \cdot \exp(\overline{g}_{I,1}/\gamma)}\right)\right]\right] + V_0 + E_L && (17)\\
& && (18)
\end{aligned}
$$

and

$$
\begin{aligned}
\mathrm{E_D}\left[\overline{V}_{D,\text{off}}\right] &= 30 \cdot \left[1 + \mathrm{E_D}\left[\tanh\left(\frac{f_E(\overline{g}_{I,2}) - b_g \cdot (g_{L,D} + \overline{g}_{I,1})}{k \cdot \exp(\overline{g}_{I,1}/\gamma)}\right)\right]\right] + V_0 + E_L && (19)\\
& && (20)
\end{aligned}
$$

We assumed that each dendrite is targeted strictly by $N_{\text{SOM}\to\text{dend}}$ SOM neurons, and since we are keeping the total amount of inhibition $G_{\text{SOM}\to\text{dend}}$ received by each dendrite fixed, the time-averaged conductance of each connection is $G_{\text{SOM}\to\text{dend}}/N_{\text{SOM}\to\text{dend}}$. We also assumed that each SOM neuron gets suppressed with probability $1-p$. Then the number of non-suppressed SOM neurons targeting each dendrite $n_{\text{SOM}\to\text{dend}}$ follows a binomial distribution

$$n_{\text{SOM}\to\text{dend}} \sim \text{B}(N_{\text{SOM}\to\text{dend}}, p) \tag{21}$$

And

$$\overline{g}_{I,1} = G_{\text{SOM}\to\text{dend}}/N_{\text{SOM}\to\text{dend}} \cdot n_{\text{SOM}\to\text{dend}} \tag{22}$$

Therefore $N_{\text{SOM}\to\text{dend}}$ determines the distribution for $\overline{g}_{I,1}, \overline{g}_{I,2}, \overline{g}_{E,1}, \overline{g}_{E,2}, \text{E}_\text{D}\left[\overline{V}_{D,\text{on}}\right], \text{E}_\text{D}\left[\overline{V}_{D,\text{off}}\right]$, and finally the gating selectivity. In summary, in the limit of a large number of dendrites, we have shown that gating selectivity only depends on the parameter $N_{\text{SOM}\to\text{dend}}$.

2. Gating selectivity strictly improves with somatic inhibition

Denote the f-I response function of the somatic compartment as $f(\cdot)$, and assume the dendritic input current to the soma is I_{on} and I_{off} when the gate is open or closed respectively. Also denote the somatic inhibitory current as I_{PV}. For convenience, assume $I_{\text{PV}} > 0$, so the outputs of the pyramidal neuron are

$$r_{\text{on}} = f(I_{\text{on}} - I_{\text{PV}}) \tag{23}$$

$$r_{\text{off}} = f(I_{\text{off}} - I_{\text{PV}}) \tag{24}$$

respectively. We consider only the case when $r_{\text{on}}, r_{\text{off}} > 0$, which means input stimuli have a net excitatory effect. Also we have $I_{\text{PV}} < I_{\text{off}}$. Since $r_{\text{on}}, r_{\text{off}}$ are baseline corrected, we should have $f(0) = 0$. Here we derive the necessary and sufficient condition for gating selectivity

$$S = \frac{r_{\text{on}} - r_{\text{off}}}{r_{\text{on}} + r_{\text{off}}} \tag{25}$$

to strictly increase with I_{PV}.

We have

$$\frac{\partial S}{\partial I_{\mathrm{PV}}} \tag{26}$$

$$= \frac{\partial}{\partial I_{\mathrm{PV}}}\left[\frac{r_{\mathrm{on}}-r_{\mathrm{off}}}{r_{\mathrm{on}}+r_{\mathrm{off}}}\right] \tag{27}$$

$$= \frac{1}{(r_{\mathrm{on}}+r_{\mathrm{off}})^2} \cdot [(r_{\mathrm{on}}+r_{\mathrm{off}})\frac{\partial}{\partial I_{\mathrm{PV}}}(r_{\mathrm{on}}-r_{\mathrm{off}}) - (r_{\mathrm{on}}-r_{\mathrm{off}})\frac{\partial}{\partial I_{\mathrm{PV}}}(r_{\mathrm{on}}+r_{\mathrm{off}})] \tag{28}$$

$$= \frac{2}{(r_{\mathrm{on}}+r_{\mathrm{off}})^2} \cdot [r_{\mathrm{off}}\frac{\partial r_{\mathrm{on}}}{\partial I_{\mathrm{PV}}} - r_{\mathrm{on}}\frac{\partial r_{\mathrm{off}}}{\partial I_{\mathrm{PV}}}] \tag{29}$$

So

$$\frac{\partial S}{\partial I_{\mathrm{PV}}} < 0 \tag{30}$$

is equivalent to

$$r_{\mathrm{off}}\frac{\partial r_{\mathrm{on}}}{\partial I_{\mathrm{PV}}} < r_{\mathrm{on}}\frac{\partial r_{\mathrm{off}}}{\partial I_{\mathrm{PV}}} \tag{31}$$

In a few more steps, we can easily derive that the necessary and sufficient condition for gating selectivity to improve with somatic inhibition is that

$$(f'(I))^2 - f(I)\cdot f''(I) > 0 \quad , \forall I > 0 \tag{32}$$

where $f'(I) = \frac{\mathrm{d}f(I)}{\mathrm{d}I}$.

We can easily see that for any power law function $f(I) = aI^b$,

$$(f'(I))^2 - f(I)\cdot f''(I) = (abI^{b-1})^2 - aI^b ab(b-1)I^{b-2} \tag{33}$$

$$= a^2 bI^{2b-2} \tag{34}$$

is strictly larger than 0, as long as $b > 0$.

References

[1] Branco, T., Clark, B. A. & Häusser, M. Dendritic discrimination of temporal input sequences in cortical neurons. *Science* **329**, 1671–5 (2010).

[2] Shoemaker, P. A. Neural bistability and amplification mediated by NMDA receptors: Analysis of stationary equations. *Neurocomputing* **74**, 3058–3071 (2011).

[3] Sanders, H., Berends, M., Major, G., Goldman, M. S. & Lisman, J. E. Nmda and gabab (kir) conductances: the "perfect couple" for bistability. *J Neurosci* **33**, 424–9 (2013).

[4] Nevian, T. & Sakmann, B. Spine Ca2+ signaling in spike-timing-dependent plasticity. *J Neurosci* **26**, 11001–13 (2006).

[5] Lee, S., Hjerling-Leffler, J., Zagha, E., Fishell, G. & Rudy, B. The largest group of superficial neocortical GABAergic interneurons expresses ionotropic serotonin receptors. *J Neurosci* **30**, 16796–808 (2010).

[6] Meyer, H. S. *et al.* Inhibitory interneurons in a cortical column form hot zones of inhibition in layers 2 and 5a. *Proc Natl Acad Sci U S A* **108**, 16807–12 (2011).

[7] Gentet, L. J. *et al.* Unique functional properties of somatostatin-expressing GABAergic neurons in mouse barrel cortex. *Nat Neurosci* **15**, 607–12 (2012).

[8] Pfeffer, C. K., Xue, M., He, M., Huang, Z. J. & Scanziani, M. Inhibition of inhibition in visual cortex: the logic of connections between molecularly distinct interneurons. *Nat Neurosci* **16**, 1068–76 (2013).

[9] Fino, E. & Yuste, R. Dense inhibitory connectivity in neocortex. *Neuron* **69**, 1188–203 (2011).

[10] Larkman, A. U. Dendritic morphology of pyramidal neurones of the visual cortex of the rat: III. spine distributions. *J Comp Neurol* **306**, 332–43 (1991).

[11] Elston, G. N., Benavides-Piccione, R. & Defelipe, J. A study of pyramidal cell structure in the cingulate cortex of the macaque monkey with comparative notes on inferotemporal and primary visual cortex. *Cereb Cortex* **15**, 64–73 (2005).
